# Quantification of Triple Single-Leg Hop Test Temporospatial Parameters: A Validated Method Using Body-Worn Sensors for Functional Evaluation after Knee Injury

**DOI:** 10.3390/s20123464

**Published:** 2020-06-19

**Authors:** Niloufar Ahmadian, Milad Nazarahari, Jackie L. Whittaker, Hossein Rouhani

**Affiliations:** 1 Department of Mechanical Engineering, University of Alberta, Edmonton, AB T6G 1H9, Canada; na2@ualberta.ca (N.A.); nazaraha@ualberta.ca (M.N.); 2 Department of Physical Therapy, University of British Columbia, Vancouver, BC V6T 1Z3, Canada; jackie.whittaker@ubc.ca; 3 Arthritis Research Canada, Richmond, BC V6X 2C7, Canada

**Keywords:** knee assessment, inertial measurement unit, ambulatory monitoring, criterion-related validation, construct validation, functional test, return-to-sport testing, triple single-leg hop test, temporal events, hopped distance

## Abstract

Lower extremity kinematic alterations associated with sport-related knee injuries may contribute to an unsuccessful return to sport or early-onset post-traumatic osteoarthritis. Also, without access to sophisticated motion-capture systems, temporospatial monitoring of horizontal hop tests during clinical assessments is limited. By applying an alternative measurement system of two inertial measurement units (IMUs) per limb, we obtained and validated flying/landing times and hop distances of triple single-leg hop (TSLH) test against motion-capture cameras, assessed these temporospatial parameters amongst injured and uninjured groups, and investigated their association with the Knee Injury and Osteoarthritis Outcome Score (KOOS). Using kinematic features of IMU recordings, strap-down integration, and velocity correction techniques, temporospatial parameters were validated for 10 able-bodied participants and compared between 22 youth with sport-related knee injuries and 10 uninjured youth. With median (interquartile range) errors less than 10(16) ms for flying/landing times, and less than 4.4(5.6)% and 2.4(3.0)% of reference values for individual hops and total TSLH progression, differences between hopping biomechanics of study groups were highlighted. For injured participants, second flying time and all hop distances demonstrated moderate to strong correlations with KOOS Symptom and Function in Daily Living scores. Detailed temporospatial monitoring of hop tests is feasible using the proposed IMUs system.

## 1. Introduction

Sport-related knee injuries, such as anterior cruciate ligament (ACL) tears, can have a significant impact on an athlete’s life. In the short-term, these injuries can lead to missed sport participation, reduced muscle strength, and increased risk of re-injury, while in the long-term, they are associated with increased adiposity, cartilage morbidity, and premature radiographic osteoarthritis [1,2,3]. Approximately 35% of athletes fail to resume previous activity levels by two years following an ACL reconstruction, and this number grows to 50% at five years post-surgery [1,4]. According to Xergia et al. [5,6], kinematic and kinetic deficiencies of the lower limbs may continue even after return to sport (RTS) and symmetrical restoration of muscular strength. These deficiencies can alter knee joint loading, which is associated with early radiographic knee osteoarthritis and cartilage degeneration [2,7]. Close monitoring of lower limb biomechanics during functional activities may assist clinical decision making related to RTS and prevention of osteoarthritis.

Functional tests, such as vertical and horizontal bilateral or unilateral jumping tests, demand muscle strength and neuromuscular coordination for dynamic joint stability, which deteriorates with a knee injury [5,8,9]. The triple single-leg hop (TSLH) test requires controlled and consecutive unilateral hops to move the body center of mass in horizontal and vertical directions without pauses, and simulates functional challenges consistent with sport maneuvers. In distance-based hop tests, the Limb Symmetry Index (LSI) of total distance hopped is a common outcome to inform RTS and rehabilitation program [10], and is defined as the ratio of distance hopped on the injured leg to the distance hopped on the uninjured leg, expressed as a percentage. In contrast, for timed hop tests, the inverse of this ratio is used to define LSI based on the time required to complete the task (i.e., uninjured leg hopping time over injured leg hopping time) [3]. Commonly, an LSI cut-off value of 85% [11,12,13] is used as a milestone for RTS clearance. Despite this standard, it is important to highlight that symmetry in total hop time or distance does not guarantee unimpaired or symmetrical hopping biomechanics [14,15].

Temporospatial parameters of functional tests have been extensively assessed in laboratories using motion-capture cameras, force platforms, and contact mattresses [16,17] to better understand the biomechanical alterations after knee injuries. These assessments are not feasible in clinical or sport training environments, given the sophisticated equipment, specialized operators, and time-consuming calibration/preparation and data post-processing required. Inertial measurement units (IMUs) have been used to obtain stride length [18,19,20] and temporal events of gait [21,22,23,24] with relatively high accuracy and precision for injured and uninjured participants. Recently, the IMUs have also been recognized as promising tools for temporospatial analyses of various vertical jumping tests [25,26,27]. These studies used kinematic features of IMU signals for temporal estimations and obtained the jump height based on ballistic equations, which were proposed merely for vertical jumping. To the best of our knowledge, IMUs have not been used to detect temporal events or measure forward progression during horizontal hop tests.

Therefore, in this exploratory study, we aimed to assess whether a wearable system of IMUs can estimate the TSLH temporospatial parameters with sufficient accuracy and precision to highlight the trend of differences in hopping kinematics of injured and uninjured leg groups during TSLH. Furthermore, it was explored whether the estimated temporospatial parameters are associated with clinically relevant patient-reported outcome scores. To these ends, a wearable system of two IMUs per limb is presented to estimate TSLH temporospatial parameters (i.e., foot–ground Initial Contact (IC) instants, foot–ground Terminal Contact (TC) instants, flying/landing times, and foot forward progression distances). First, the criterion-related validity of these IMU-based temporospatial parameters was assessed against reference values obtained with motion-capture cameras. Specifically, we compared the accuracy and precision of the IMU-based temporospatial parameters of a TSLH test to the criterion-standard motion-capture cameras to determine whether the IMU-based system is a feasible substitute. Second, the construct validity of the IMU-based system was assessed by comparing TSLH kinematics between youth who had experienced a sport-related intra-articular knee injury, and likely to demonstrate kinematic alterations and uninjured active youth who are not likely to demonstrate kinematic alterations. This involved comparing intra-participant (side-to-side) and inter-participant (injured versus uninjured group) temporospatial parameters and corresponding LSIs. IMU-based estimates of total and LSI TSLH progression were further validated by comparing them to results obtained with a measuring tape (clinical standard) and assessing their association with self-reported knee-related symptoms and function (Knee Injury and Osteoarthritis Outcome Score subscales; KOOS) in injured participants.

## 2. Materials and Methods

**Figure 1:**
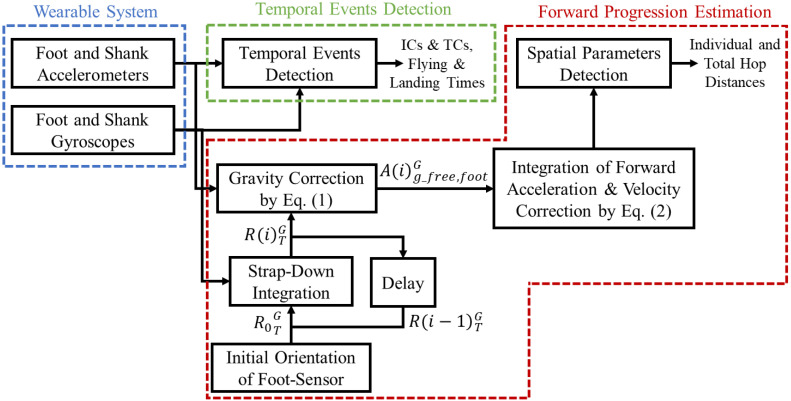
The flowchart of the proposed procedure for detecting triple single-leg hop (TSLH) temporospatial parameters using inertial measurement units (IMUs). Each step is described in detail in the sections distinguished with dashed lines. The box labelled as “Delay” brings the newly calculated foot-sensor orientation at the current time instant back to the strap-down integration box to estimate the foot-sensor orientation in the next time instant.

The proposed procedure for detecting TSLH temporospatial parameters using IMUs is shown in Figure 1. The wearable system is described in Section 2.1, the temporal event detection algorithm is detailed in Section 2.4.2, and the forward progression estimation is described in Section 2.5.2. The criterion-related validation of TSLH temporospatial parameters measured by the proposed wearable system, and construct validation in patients compared to controls is described in Section 2.3.1 and Section 2.3.2, respectively.

### 2.1. Wearable Measurement System

**Figure 2:**
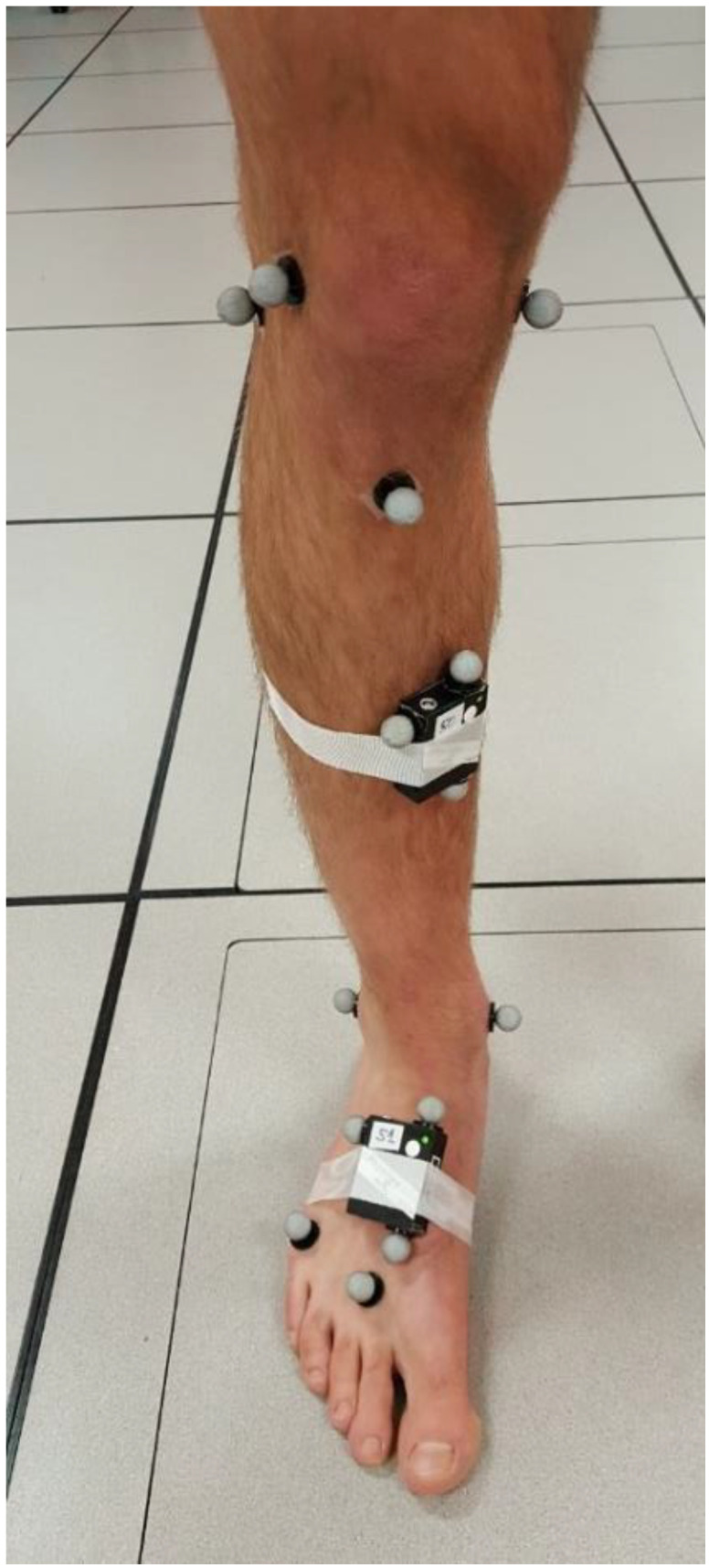
Illustration of the criterion-related validation set-up: Anatomical (applied to bony landmarks) and technical (applied to sensors boxes) markers, as well as the two IMUs, affixed on the right foot (along the second metatarsal) and leg (medial upper shank) of the participant, are represented. Among the anatomical markers, only the second and fifth metatarsal, and calcaneal tuberosity markers were used for this study. No marker was present for the construct validation study.

To investigate the criterion-related validity of the proposed temporospatial measurements, two IMU modules (Physilog BFSr-3, Gait Up, Lausanne, Switzerland, weight: 36 grams) were affixed with single- and double-sided hypoallergenic tapes to the dominant lower extremity of participants at forefoot and upper shank regions (Figure 2). These modules wirelessly recorded 3D acceleration (range: ±11 g) and 3D angular velocity (range: ±1200 °/s) with the sampling frequency of 500 Hz.

To investigate the construct validity of the outcome measures, four modules of lighter, recently released IMUs (Physilog 5, Gait Up, Lausanne, Switzerland, weight: 11 grams) were bilaterally attached with Velcro straps to the feet and shanks of participants at regions consistent with the criterion-related validation study. These modules were set to record 3D acceleration (range: ±16 g) and 3D angular velocity (range: ±2000 °/s) with the sampling frequency of 256 Hz.

### 2.2. The Reference System

The positions of two sets of anatomical and technical reflective markers, as shown in Figure 2, were tracked with 8 motion-capture cameras (Motion Analysis Corporation, Santa Rosa, CA, USA) and used as the reference to detect temporal events and foot forward progression during TSLH trials. Anatomical markers in Figure 2 were placed on the second and fifth metatarsal heads, and calcaneal tuberosity through palpation, following the well-known protocol in [28] for foot segment position and orientation tracking. Three technical markers (see Figure 2) were attached to each of the IMU boxes and were used for reconstruction of anatomical markers, where they were absent. Marker positions were originally recorded at 100 Hz synchronously with the IMU recordings and up-sampled (using linear interpolation) to 500 Hz during the analyses for comparison with the IMU-based system. The power spectral density graph of the raw marker data showed that almost all of the frequency contents of the time-series for 3D coordinates of all three technical markers’ trajectories were in the range of 0 to 5 Hz. Therefore, up-sampling these data, using linear interpolation, did not affect the analyses.

### 2.3. Experimental Protocol

#### 2.3.1. Criterion-Related Validation Experiments

Through advertising on the University of Alberta campus, ten able-bodied men with no history of severe knee injuries or musculoskeletal disease volunteered to participate in the study. With the application of both IMU-based and reference systems, the participants were asked to stand motionlessly in their natural posture for 1 minute while the cameras recorded the position of both technical and anatomical markers indicated in Figure 2. To avoid marker-falling during the highly dynamic hop trials, the anatomical markers were then removed. These markers were later reconstructed as virtual markers based on the technical markers of the foot-sensor. In the few instances when one of the technical markers was missing for a few frames, it was reconstructed using “pattern fill” and based on the position of another technical marker on the same IMU. To reduce the effect of soft-tissue artifact on IMU data and technical markers, the IMU modules were tightly secured to the body segments using both single- and double-sided hypoallergenic tapes. The main TSLH trials started and ended with 10-second periods of standing still, and participants were asked to perform two successful TSLH trials on their dominant leg.

#### 2.3.2. Construct Validation Experiments

From an ongoing prospective cohort of 11–19-year-old youth, a convenience subsample of 22 participants with a sport-related knee injury (group G_I_), and 10 uninjured participants (group G_UI_) were outfitted with IMUs (See Section 2.1). Knee injury was defined as a clinical diagnosis of knee ligament, meniscal, or other intra-articular tibiofemoral or patellofemoral injury that needed both medical consultation and disruption in regular sports participation. While the G_I_ group had sustained a unilateral knee injury within 15 months prior to the testing date, the G_UI_ participants had no history of lower limb injuries. Prior to testing, participants’ age, bare feet standing height, body mass, and dominant leg (preferred kicking leg) were recorded. Participants also underwent a clinical physiotherapy knee examination and completed the KOOS questionnaire. The KOOS is a self-report measure designed to evaluate symptoms and function related to the knee injury and osteoarthritis in active young patients. It consists of 42 items in five subscales (Symptom, Pain, Function in Daily Living (ADL), Function in Sports and Recreation (Sport/Rec), and Knee Related Quality of Life (QoL)) which are scored on a 5-point Likert scale. Subscale scores are transformed to a 0–100 scale with higher scores indicating better function [29].

Individuals were eligible to participate in the study if they had full knee range-of-motion, no knee effusion, and no pain and difficulty with twisting/pivoting/jumping. Participants performed two successful TSLH trials with each leg, initiated and ending with 5-seconds of upright motionless posture. A successful TSLH trial was defined as a trial in which the three hops were performed consecutively with a controlled landing after the last hop in which no extra hops or considerable ankle twists were involved. For each successful TSLH trial, the total forward progression was recorded with a measuring tape [9]. The ethics board of the University of Alberta approved both studies (Pro00065804 and Pro00063773), and the participants and/or their guardian provided informed written consent and/or assent prior to participation.

### 2.4. Temporal Events Detection

#### 2.4.1. Reference Temporal Events

Unlike gait, where heel-strike precedes toe-strike, in the TSLH, any foot region can be the site of initial contact (IC) and terminal contact (TC). Therefore, all anatomical foot markers shown in Figure 2 were reconstructed as virtual markers, assuming that the foot moves as a rigid body [30]. Before any data processing, signals of the reconstructed foot markers were low-pass filtered using a zero-phase 4th-order digital Butterworth filter with a cut-off frequency of 20 Hz. The average height of each reconstructed marker during the starting motionless period was considered as a reference level. Time frames of intersections between the recorded marker heights and their corresponding reference levels were defined as IC and TC events [31,32,33]. Due to abrupt changes of marker heights close to IC instants, resulting from sensor wobbling at the time of foot–ground impact, the IC detection method was further refined by shifting the previously detected approximate ICs forward, to the next minimum of marker height recordings. In addition to ICs and TCs, we defined three flying times (the period where the foot was not in contact with the ground) as the time interval between each IC and the last TC, and two landing times (the period where at least one region of the foot was in contact with the ground) as the time interval between each TC and the last IC.

#### 2.4.2. IMU-based Temporal Events

Peak detection algorithms for foot and shank angular velocity and acceleration signals were introduced similar to [21,22,23] and compared to the reference temporal instants. Methods provided by [21,22] for gait temporal events detection were modified and adapted to hopping kinematics. Likewise, all gait temporal features investigated by [23] were assessed for recordings of both foot and shank IMUs, during TSLH. Additionally, due to their relevance to hopping events and their independence to the IMUs orientation on the foot or shank, the absolute values of time-derivative of acceleration norm signal for foot and shank ($\left|\|A_{F}\|{}^{\prime }\right|$ and $\left|\|A_{S}\|{}^{\prime }\right|$) were assessed.

**Figure 3:**
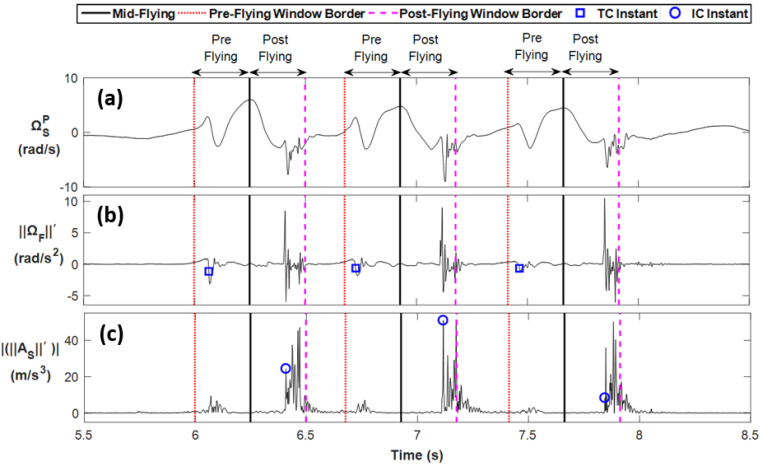
Terminal Contact (TC) and Initial Contact (IC) instants of a typical TSLH trial, detected using IMUs. (**a**): Mid-flying instants (solid vertical lines) are estimated based on shank pitch angular velocity signal ($\Omega_{S}^{P}$). Pre-flying (dotted vertical lines) and post-flying (dashed vertical lines) phases are defined 250 ms prior and after the mid-flying instants, respectively. (**b**): TC instants (shown with squares) are marked on the time-derivative of foot angular velocity norm time-series ($\|\Omega_{F}\|{}^{\prime }$). (**c**): IC instants (shown with circles) are marked on the absolute value of time-derivative of shank acceleration norm time-series *(*$\left|\|A_{S}\|{}^{\prime }\right|$).

The global peak of shank pitch angular velocity ($\Omega_{S}^{P}$) at each hop cycle was used as a robust feature to detect mid-flying instants [22,23] and to split each hop cycle into pre-flying and post-flying time windows for TC and IC detection (Figure 3). We found the best IC-related feature to be the first peak of $\left|\|A_{S}\|{}^{\prime }\right|$, having an amplitude greater than 7 m/s^3^ and occurring no later than 250 ms after the mid-flying instant of each hop cycle. Searching the pre-flying window of length 250 ms, we found the best TC-related feature based on the time-derivative of foot angular velocity norm, ($\|\Omega_{F}\|{}^{\prime }$), at the time when its amplitude falls below −0.6 rad/s^2^.

### 2.5. Forward Progression Estimation

#### 2.5.1. Reference Forward Progression

The unfiltered data of the reference motion-capture system were used for forward trajectory measurement. The average trajectory of the foot IMU markers in Figure 2 was considered representative of the foot trajectory. Forward progression at each hop cycle was defined as the foot frontal progression between $mqs_{n-1}$ and $mqs_{n}$, where $mqs_{n}$ marks the middle of the quasi-stance phase after the *nth* hop cycle. The quasi-stance phases were considered as the largest interval between ICs and the next consecutive TCs, where the norm of the foot angular velocity was always less than 4 rad/s.

#### 2.5.2. IMU-based Forward Progression

To obtain the forward progression during TSLH, the gravity-free acceleration of the foot in the lab global frame was double integrated, and a corrective function was defined at each hop cycle to remove the foot velocity drift caused by integration [18,19]. The gravity-free foot acceleration was calculated as in Equation (1):

(1) $$A{\left(i\right)}_{g\_free,foot}^{G}=R_{0}{}_{T}^{G}\;{\bar{g}}^{T}-R{\left(i\right)}_{T}^{G}\;A{\left(i\right)}_{raw,foot}^{T},$$

where $A{\left(i\right)}_{g\_free,foot}^{G}$ and $A{\left(i\right)}_{raw,foot}^{T}$ denote the gravity-free acceleration of foot in the lab global frame and the foot-accelerometer readout in its technical frame at each time sample (*i*), respectively. $R_{0}{}_{T}^{G}$ represents the rotation matrix between the foot-sensor technical frame and the lab global frame during the static stance period prior to the hopping and can be calculated based on the position of foot-sensor technical markers in Figure 2. In the construct validation study, where the markers were not present, $R_{0}{}_{T}^{G}$ was obtained using the accelerometer readout [34]. ${\bar{g}}^{T}$ shows the median of foot-accelerometer readout in sensor technical frame during the static stance, with the assumption that it is caused solely by gravity. $R{\left(i\right)}_{T}^{G}$ denotes the rotation matrix between the foot-sensor technical frame and the lab global frame at each time sample, (*i*), and is calculated based on the strap-down integration of the de-drifted foot angular velocity signal, as described in [18,19,35].

A corrective piecewise cubic Hermite interpolating polynomial (p-chip function) was subtracted from the forward component of trapezoidal integration of $A{\left(i\right)}_{g\_free,foot}^{G}$ to derive foot forward velocity. This sigmoid-like corrective function was defined over $\left[mqs_{n-1},mqs_{n}\right]$, with the axillary point $aqs_{n}$ at 75% of this interval, as in Equation (2):

(2) $$f_{c}(n):=pchip\left(\left\{mqs_{n-1},aqs_{n},mqs_{n}\right\},\left\{V_{F}\left(mqs_{n-1}\right),min\left(V_{F}\left(mqs_{n-1}\right),V_{F}\left(mqs_{n}\right)\right),V_{F}\left(mqs_{n}\right)\right\}\right),$$

where $f_{c}\left(n\right)$ and $V_{F}$ denote the defined corrective function and the non-corrected foot forward velocity, respectively. Finally, foot forward progression was derived from the integration of corrected foot forward velocity.

### 2.6. Data Analysis and Statistical Tests

For criterion-related validation, 60 hop cycles (10 participants × 2 trials × 3 hops at each trial) were investigated, and the temporospatial parameters obtained by IMUs were compared to those obtained by the reference system. Medians and interquartile ranges of errors were considered as accuracies and precisions of the IMU-based temporospatial parameters. For construct validation, 132 hop cycles (22 participants × 2 trials × 3 hops) and 60 hop cycles (10 participants × 2 trials × 3 hops) were investigated for each leg of G_I_ and G_UI_, respectively. For all participants, three hopping forward distances, total TSLH forward progression, three flying and two landing times, and corresponding LSIs were calculated based on IMU signals. The averaged temporospatial parameters over the two trials recorded from each leg, were considered for statistical analysis. As the normality of the samples was rejected for a number of investigated parameters using Jarque–Bera test, Wilcoxon signed-rank test and Wilcoxon rank-sum test were used for intra-participant (side-to-side) and inter-participant (injured/uninjured legs of G_I_ compared to dominant/non-dominant/both legs of G_UI_) comparisons, respectively (*α* = 0.05). Clinical relevance of the obtained temporospatial parameters and LSIs was further explored by calculating their Spearman’s correlation with G_I_’s KOOS subscale scores. Correlation coefficients were interpreted as very weak (0≤|r|≤0.19), weak (0.2≤|r|≤0.39), moderate (0.4≤|r|≤0.59), strong (0.6≤|r|≤0.79), and very strong (0.8≤|r|≤1) [36].

## 3. Results

### 3.1. Estimations of Temporospatial Parameters: IMU Versus Reference Systems

For the criterion-related validation trials (see Table 1 for participant characteristics), the proposed kinematic features of IMU signals estimated the IC and TC instants with median (interquartile range: IQR) errors of 2 (22) and 12 (24) ms, respectively, compared to the reference system. These errors in IC and TC detection were equal to 0(2) and 1(2) time samples of the reference system recordings, respectively. Flying (60 flying phases) and landing (40 landing phases) times were estimated with median (IQR) differences of −4(18) and 6(18) ms from the reference values (i.e., 0(2) and 1(2) time sample of the reference system recordings). It should be noted that both IMUs and motion-capture systems’ data were analyzed with a sampling frequency of 500 Hz; thus, the resolution for presenting errors in temporal events detection is 2 ms. Also, following the common representation of errors for temporal events detection similar to gait studies, we used both positive and negative errors to demonstrate the direction of the bias in the IMU system with respect to the reference motion-capture system [23,32,33]. Individual hop distances (60 hops) were estimated with 4.4%(5.6%) relative error while the total TSLH progression (20 TSLH trials) were estimated with 2.4%(3.0%) relative error compared to the reference system (Table 2). In the construct validation study, comparison of the IMU-based total TSLH forward progression with the values obtained with a measuring tape showed median (IQR) relative errors of 2.9%(3.5%) and 3.2%(2.9%) (LSIs absolute errors of 4.8%(3.0%) and 3.9%(2.1%)) for G_I_ and G_UI_, respectively.

### 3.2. Comparison of Temporospatial Results among Injured and Uninjured Youth

No significant between-group differences were observed for height and body mass (see Table 1 for participant characteristics); however, G_I_ was significantly younger than G_UI_ (*p* = 0.007). No significant differences (*α* = 0.05) were observed in any of the inter- and intra-participant comparisons of IMU-based temporospatial parameters and LSIs, except for the first landing time (*p* = 0.049 between the non-injured side of G_I_ and both sides of G_UI_). Total TSLH progression measured with a measuring tape showed a significant difference (*p* = 0.028) for the intra-participant comparison of the G_I_. No other statistically significant differences were found between the compared temporospatial parameters or LSI values.

**Figure 4:**
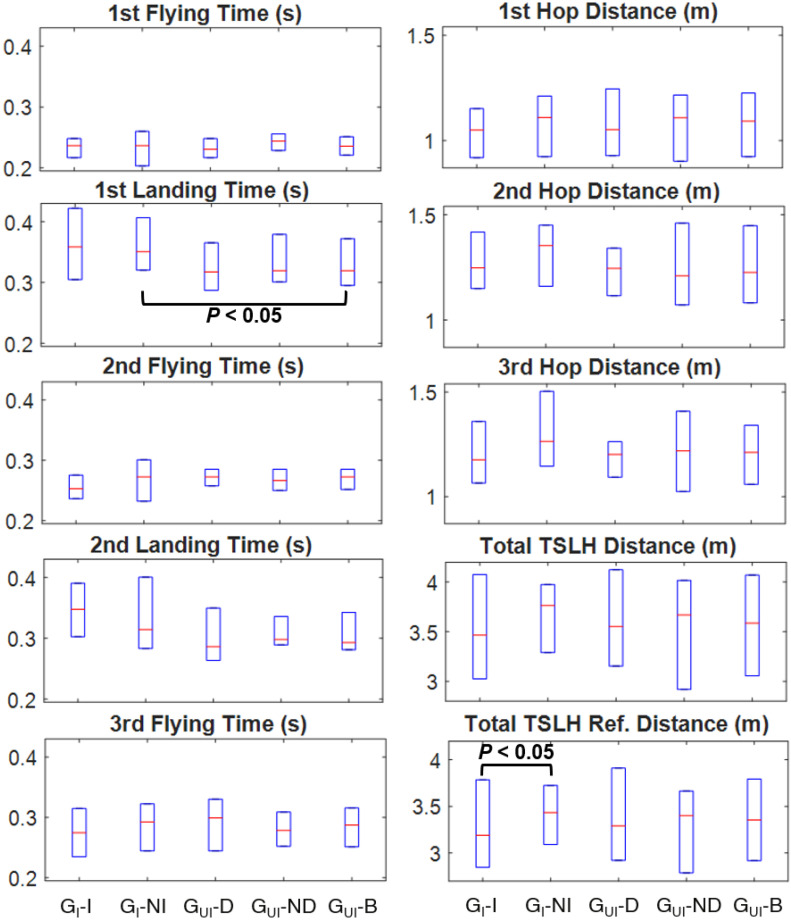
Comparison of temporospatial parameters among all different leg sub-groups of injured (G_I_) and uninjured (G_UI_) participants. Temporospatial parameters were estimated with IMUs or measured with a measuring tape (shown as Total TSLH Ref. Distance). Box-plots on the left and right sides represent time- and distance-related parameters, respectively. (Leg sub-groups are shown with G_I_-I: G_I_’s Injured Leg, G_I_-NI: G_I_’s Non-Injured Leg, G_UI_-D: G_UI_’s Dominant Leg, G_UI_-ND: G_UI_’s Non-Dominant Leg and G_UI_-B: G_UI_’s Both Legs.).

Non-statistically significant differences of temporospatial parameters of the injured/non-injured sides of G_I_, and dominant/non-dominant/both sides of *G*_UI_ were observed (Figure 4). Specifically, the non-injured side of G_I_ had the greatest median individual and total hop distance values among all the leg sub-groups, while the injured side had the lowest median distance value, except for the second hop. Additionally, the injured side of G_I_ had the longest median landing and shortest median flying time values of all leg sub-groups, except for the first flying time. Finally, the dominant side of G_UI_ had the shortest median landing and longest median flying time values of all leg sub-groups, except for the first flying. However, none of the three sets of comparisons mentioned above were statistically significant.

### 3.3. Correlations between the KOOS and Temporospatial Parameters in Injured Youth

Based on both measuring systems (IMU and measuring tape), all individual and total hop distances of the G_I_’s injured side were moderately correlated with the KOOS Symptom subscale score (0.403 < *r* < 0.502) (Table 3). All individual and total hop distances of G_I_’s injured side except for the third hop distance also were moderately correlated with the KOOS Function in Daily Living (ADL) subscale score (0.407 < *r* < 0.429). The KOOS Symptom and ADL scores also were moderately correlated with LSI values of total TSLH distance measured with tape (*r* = 0.414) and IMU-based second hop distance LSI (*r* = −0.407), respectively. Among the temporal parameters, the second flying time showed strong (*r* = 0.660) and moderate (*r* = 0.448) correlation with the KOOS Symptom and ADL subscale scores, respectively. No moderate or strong correlation was observed between temporal LSIs and KOOS scores, except for the LSI calculated based on second flying time and KOOS Symptom score (*r* = −0.536). Generally, the correlations of both KOOS Symptom and ADL scores were considerably stronger with flying times than landing times (Table 3). Nearing the end of TSLH trials, correlations increased between the distance hopped and the KOOS Symptom subscale, while the opposite trend was observed for KOOS ADL subscale. All other KOOS subscale scores had a very weak or weak correlation with the calculated temporospatial parameters and LSIs except for a correlation between second flying time and KOOS Function in Sports and Recreation subscale score (*r* = 0.401), and correlations of second hop distance LSI with KOOS Pain subscale (*r* = −0.402) and KOOS Quality of Life subscale scores (*r* = −0.545).

## 4. Discussion

In this study, IMUs were used for the first time to calculate the forward progression and flying/landing periods during each phase of the TSLH test, and the results were validated against a reference system of motion-capture cameras. The efficacy of the introduced temporospatial measuring system was then explored in a clinical research environment for intra-participant and inter-participant comparisons between groups of uninjured and injured youth. Such a system can be used to break down the horizontal hop tests into their sub-phases and measure the temporospatial parameters along the multiple horizontal hops. To the authors’ knowledge, it is the first time that the jumping/hopping distance during the functional tests has been calculated directly based on the acceleration of body segments rather than the flying time.

Our proposed algorithm was able to detect IC and TC instants of the TSLH tests with median errors of less than 12 ms (i.e., one time sample of the reference system recordings). As expected, the error of TC detection was slightly higher than IC, as TCs are smoother temporal events, and unlike ICs, they are not associated with abrupt changes and peaks in IMU recordings. These errors were comparable to those of [21,22,23] obtained for temporal parameters of gait. However, notably larger errors are expected in the detection of temporal parameters due to the jerky motion of the foot in the TSLH test, accompanied by severe IMUs wobbles. Compared to vertical jumping studies [25,27], we obtained similar accuracy and precision in temporal parameters detection. However, those studies had removed the constant erroneous offsets of IC and TC estimation from their results.

The accuracy and precision of our estimated forward progression during TSLH were comparable to those reported in [19] for stride length estimation. While we estimated individual hop distances with a median error of 5.41 cm, [18] reported root-mean-square error of about 18 cm for gait progression estimation. Additionally, as shown in Table 2, the error of forward progression estimation does not increase along the test, which was to be expected according to [37] for a short test such as TSLH (duration less than 30 seconds). Therefore, removing the gyroscopes’ static drift at the beginning of the TSLH trial effectively eliminated gyroscope-based errors, and further correction for these errors was not required.

The moderate to strong correlations of several IMU-based temporospatial parameters with KOOS Symptom and ADL subscales emphasize the relevance and importance of monitoring each hop in detail during the TSLH test to interpret those scores during functionally challenging activities, rather than merely recording the total distance. The validity, responsivity, and reliability of KOOS for populations with various knee injuries have been reported by several studies [3,38,39]. Our results suggest that differences due to knee injury symptoms may be more apparent towards the end of a TSLH trial when the activity becomes more challenging, while differences associated with decreases in ADL are apparent at the time of activity initiation. As the IMU-based system can provide instantaneous temporospatial information during the TSLH test, the interpretation of KOOS subscale scores in association with temporospatial parameters would be feasible for clinical scientists. Furthermore, the consistent trend of correlations of temporospatial parameters with KOOS Symptom and ADL scores from the first to the third hop makes them reliable outcomes for clinical interpretations.

Although no significant difference was observed for IMU-based temporospatial parameters among injured and uninjured youth (possibly due to the heterogeneity of injury, variability in time since injury, and small sample size), the proposed measuring system showed consistent results with previous studies [12,17] on shorter distances hopped on G_I_’s injured leg. Although the total TSLH progression measured by tape showed a significant intra-participant difference for G_I_ (*p* = 0.028), it is not clear how the process of eyeballing the tape numbers in clinics would affect the accuracy of this method. The proposed IMU-based system was validated with the gold standard of motion-capture with a median error of 2.4% for total TSLH progression and resulted in *p* = 0.088 for G_I_’s intra-participant comparison, for which median LSI of total hop progression was 94.3%.

The proposed IMU-based system is expected to measure temporospatial parameters accurately for similar horizontal hop tests. The development of the algorithm for 3D foot trajectory estimation can provide further details for the tests such as crossover horizontal hops, where the lateral trajectory of the foot can also be of interest. Furthermore, verbally encouraging the participants to perform consecutive hops during TSLH and rejection of trials with longer pauses between the hops might conceal the tendency to longer landing times in injured participants. These longer pauses might be due to more time that injured participants need to re-coordinate their joints for the next hop and can be a result of stiff landing strategies [5,12]. As our proposed system can accurately estimate landing times, there is no need for the further implication of such standards as “hopping without pauses,” and the participants can hop at their comfortable pace. Therefore, enhancement and comparison of the existing hop tests will be feasible in the future, in order to introduce a test which can address knee deficits more comprehensively.

### Limitations and Future Directions

A number of limiting factors must be noted. First, we used a more recently released generation of IMUs in the construct validation study, with which we expected to obtain more accurate results than those used for the criterion-related validation. Despite this discrepancy, the median relative error of the estimated total hopped distance and the absolute error of its corresponding LSI were below 5% (compared to those measured by tape as a secondary reference system). This further confirmed the accuracy of the IMU system used in the construct validation study. Second, we used the technical markers to reconstruct the anatomical ones during the TSLH. This can lead to errors in the position of the reconstructed anatomical markers due to soft tissue artifact, which we tried to eliminate by tightly securing the IMU modules to the body segments. Also, to avoid violating the rigid body assumption for the foot, we only used the motion of the second and fifth metatarsal heads and calcaneal tuberosity and did not use the toe motion. In general, the higher dynamics of the hopping compared to gait can be the source of many errors such as deformation of body segments, vibrations of the soft tissue on which IMUs are attached, and increased gyroscopes’ drift due to the complicated and dynamic movements.

Third, the number of participants in the construct validation study, especially within the uninjured group, was limited. Thus, the application of the proposed wearable system must be further investigated with more participants before making any conclusion about the clinical significance of the evaluated parameters. Notably, the objective of the construct validation study was to compare individuals that are likely to demonstrate altered kinematics due to an injury and individuals that are less likely to demonstrate alterations. Given this objective, the limited sample size, between-group differences in age, and a greater number of women participants, this study should be treated as an exploratory study and no clinical conclusions should be made based on the findings. Fourth, for clinical use, further investigation should be performed on healthcare professionals’ and patients’ acceptance rate of this system and the simplicity of using this system by the personnel who are unfamiliar with the wearable systems. Fifth, force plates were not used as the gold standard of temporal events detection during criterion-related validation. This is an inherent limitation of TSLH tests in which the position of force plates cannot be predicted for each hop performed by every individual, without affecting the dynamics of hopping. To minimize the effect of this limitation, well-known validated methods were applied to motion-capture data to find the temporal events.

## 5. Conclusions

We presented a system of two IMUs fixed on shank and foot, capable of estimating foot–ground IC and TC instants, and forward progression along the horizontal hop tests such as TSLH, and demonstrated its accuracy and precision. This system can help clinician-scientists to study the detailed biomechanical parameters of hopping during rehabilitation programs, as relevant variables to clinically meaningful scores and decide about RTS onset with more confidence.

## Figures and Tables

**Table 1:** Characteristics of the participants enrolled in the criterion-related validation and construct validation studies. For the participants of the construct validation study, self-reported Knee Injury and Osteoarthritis Outcome Score (KOOS) scores are also presented. The abbreviations used in the table are as follows: n: Number of participants; W: Woman; M: Man; R: Right; L: Left; ADL: KOOS subscale for Function in Daily Living; Sport/Rec: KOOS subscale for Function in Sports and Recreation; and QoL: KOOS subscale for Knee Related Quality of Life.

Characteristics	Criterion-Related Validation (*n* = 10)		Construct Validation (*n* = 32)	
	Injured (*n* = 0)	Uninjured (*n* = 10)	Injured ^1^ (*n* = 22)	Uninjured ^1^ (*n* = 10)
**Sex (W/M)**	-	0W/10M	17W/5M	9W/1M
**Age ^2^ (years)**	-	23 ± 3	16 ± 1	17 ± 2
**Height ^2^ (cm)**	-	177 ± 10	167 ± 12	171 ± 9
**Body Mass ^2^ (kg)**	-	68 ± 8	60 ± 14	65 ± 10
**Dominant Leg (R/L)**	-	10R/0L	19R/3L	10R/0L
**Injured Leg (R/L)**	-	-	12R/10L	-
**KOOS Symptom ^2^**	-	-	84 ± 16	95 ± 6
**KOOS ADL ^2^**	-	-	99 ± 4	100 ± 0
**KOOS Pain ^2^**	-	-	90 ± 14	100 ± 0
**KOOS Sport/Rec ^2^**	-	-	85 ± 20	100 ± 0
**KOOS QoL ^2^**	-	-	50 ± 30	100 ± 5

^1^ Injured and uninjured groups of participants of the construct validation study are referred to as G_I_ and G_UI_, respectively, within the body of this article. ^2^ Age, height, body mass, and self-reported KOOS scores are presented as median ± interquartile range among the participants.

**Table 2:** Errors of the proposed IMU system in the estimation of temporospatial parameters of TSLH against the motion-capture system. Results are expressed as 25%, 50%, and 75% percentiles of error, calculated for all 60 individual hops (20 TSLH trials, 3 hops each) performed by the 10 able-bodied participants. The values in parentheses for the temporal parameters express errors in terms of the time sample of the motion-capture system (10 ms).

Validated Parameter	Error ^1^					
	(25%	50%	75%)	(25%	50%	75%)
**Temporal Parameters**	**Error ^1^** **(ms and sample)**			**Absolute Error ^1^** **(ms and sample)**		
**Initial Contact Instants**	[−10(−1)	2(0)	12(1)]	[4(0)	12(1)	20(2)]
**Terminal Contact Instants**	[−8(−1)	12(1)	16(2)]	[10(1)	14(1)	20(2)]
**Flying Times**	[−12(−1)	−4(0)	6(1)]	[6(1)	10(1)	18(2)]
**Landing Times**	[−4(0)	6(1)	14(1)]	[4(0)	10(1)	20(2)]
**Forward Progression Distances**	**Relative Error (%) ^1^**			**Absolute Error (cm) ^1^**		
**First Hops**	[3.62	5.50	6.31]	[4.46	6.11	7.17]
**Second Hops**	[1.71	3.05	6.52]	[1.90	3.82	7.54]
**Third Hops**	[2.15	5.64	8.65]	[2.39	7.39	10.78]
**All Individual Hops ^2^**	[2.08	4.44	7.69]	[2.42	5.41	9.77]
**Total TSLH Progression ^2^**	[1.03	2.40	4.01]	[3.83	9.35	14.12]

^1^ Error, Absolute Error, and Relative Error are calculated by subtracting the IMU-based temporospatial values from their corresponding motion-capture values (including the sign), taking the absolute value of the aforementioned differences, and dividing these absolute values by the motion-capture values and expressing them in the form of percentages, respectively. ^2^ Individual Hops refer to each of the three distances of hop cycles during a TSLH trial, while Total TSLH Progression indicates the total distance hopped during a TSLH trial.

**Table 3:** Spearman’s correlation coefficients between KOOS subscale scores and temporospatial parameters obtained with IMUs or measuring tape for the injured leg of the injured participants (G_I_) during all TSLH phases. Significant correlations are marked with bold numbers. The abbreviations used in the table are as follows: ADL: KOOS Function in Daily Living subscale; Sport/Rec: KOOS Function in Sports and Recreation subscale; and QoL: KOOS Knee Related Quality of Life subscale. Correlation strength was interpreted very weak (0–0.19), weak (0.2–0.39), moderate (0.4–0.59), strong (0.6–0.79), and very strong (0.8–1) [36].

**KOOS Subscale**	**Time**					**Time Asymmetry ^1^**				
	**Fly 1**	**Land 1**	**Fly 2**	**Land 2**	**Fly 3**	**Fly 1**	**Land 1**	**Fly 2**	**Land 2**	**Fly 3**
**Symptom**	0.166	0.003	**0.660**	0.055	0.381	−0.117	0.049	**−0.536**	0.245	0.016
**ADL**	0.390	−0.027	**0.448**	0.085	0.157	−0.225	0.254	−0.366	0.291	0.044
**Pain**	0.126	0.052	0.253	0.095	−0.056	−0.054	−0.014	−0.148	0.082	0.317
**Sport/Rec**	0.117	−0.035	0.401	−0.075	0.067	−0.070	0.109	−0.363	0.357	0.108
**QoL**	0.001	−0.150	−0.062	−0.005	−0.158	−0.249	0.338	−0.137	0.328	−0.121
**KOOS Subscale**	**Distance**					**Distance Asymmetry ^2^**				
	**Hop 1**	**Hop 2**	**Hop 3**	**TSLH Total**	**TSLH Total (Ref.) ^3^**	**Hop 1**	**Hop 2**	**Hop 3**	**TSLH Total**	**TSLH Total (Ref.) ^3^**
**Symptom**	0.403	**0.485**	**0.502**	**0.494**	**0.502**	0.259	−0.201	0.312	0.177	0.414
**ADL**	**0.429**	0.414	0.327	0.407	**0.422**	0.178	−0.407	0.181	0.024	0.324
**Pain**	0.245	0.175	0.206	0.222	0.253	0.115	−0.402	0.137	−0.044	0.170
**Sport/Rec**	0.308	0.259	0.157	0.273	0.263	0.168	−0.363	0.087	−0.039	0.231
**QoL**	0.119	0.203	0.222	0.151	0.239	−0.041	−0.545	0.071	−0.156	0.059

^1^ Time-based LSIs (in percentage) were calculated for each hop phase as the ratio of the time that the participants needed to hop on their non-injured leg over the time that they needed to hop on their injured leg. ^2^ Distance-based LSIs (in percentage) were calculated as the ratio of distance that participants have hopped with their injured leg over the distance they have achieved with their non-injured leg. ^3^ TSLH Total (Ref.): Reference values of total hopped distance, measured with a measuring tape.
